# Utilizing the Intercultural Development Inventory® to develop intercultural competence

**DOI:** 10.1186/2193-1801-3-334

**Published:** 2014-07-01

**Authors:** Julie A Kruse, Judy Didion, Kathy Perzynski

**Affiliations:** College of Nursing, Lourdes University, 6832 Convent Boulevard, Sylvania, OH 43560 USA

**Keywords:** Cultural development, Intercultural development inventory, Cultural development framework, Cultural development in nursing education, Nursing workforce diversity

## Abstract

**Purpose:**

Health care professional education programs in the United States have been charged to devise strategies to increase the racial and ethnic diversity of the workforce (Health Resources and Services Administration, Nursing Workforce Diversity (NWD) http://bhpr.hrsa.gov/nursing/grants/nwd.html, 2014). The purpose of this charge is to develop a healthcare workforce that can better provide culturally relevant care to meet the needs of diverse communities. The purpose of this study was to assess the cultural competency of students, faculty, and staff from a small Midwest-university college of nursing.

**Methods:**

This study was part of a larger interventional study to enhance the cultural development of the College of Nursing faculty, staff, and students. The sample for this study included 314 participants (students, faculty, and staff) in phase one of the parent study. Phase one included the initial administration of the Intercultural Development Inventory (IDI®) over a two year period with analysis of the pre-test results. Phase two includes the implementation of cultural development interventions with a post-test IDI® survey and is currently in process.

**Results:**

IDI® aggregate results were similar for students and faculty/staff in that most participants scored at the Minimization level according to the IDI®. Ninety-eight percent of student participants overestimated their level of cultural competency. Minority students had higher cultural competency scores in terms of developmental orientation (M = 98.85, SD = 14.21) compared to non-minority students (M = 94.46, SD = 14.96).

**Conclusions:**

Overall, the IDI® was a valuable self-reflection tool to assess cultural development. At the individual level, it has allowed for self-reflection and awareness to the reality of cultural development, attitudes, and values. At an institutional level, the aggregate results provided a framework for the examination of department policies, procedures, and curriculum design with the ultimate goal of graduating a more culturally competent nursing workforce to serve the greater community.

## Introduction

Health care professional education programs in the United States have been charged to devise strategies to increase the racial and ethnic diversity of the workforce (Health Resources and Services Administration 2014). The purpose of this charge is to develop a healthcare workforce that can better provide culturally relevant care to meet the needs of diverse communities. The Health Resources and Services Administration (HRSA), Bureau of Health Professions, Division of Nursing prioritized this purpose with an appeal for educational interventions that include a strong focus on cultural competence-building with faculty, staff, and students to increase the diversity of the nursing workforce from local to national levels.

The reason for this emphasis is that underserved populations face special challenges in both disconnects and discrimination as reported by Villarruel et al. 2014. The project described in this paper answers this charge not only through emphasis on diversity in curriculum development but also by developing strategies to recruit and retain a diverse student nurse population. One of the retention efforts was to improve the cultural competency of faculty, staff, and the student body. The Intercultural Development Inventory (IDI®) is one method to assess cultural sensitivity and competence. This instrument was deployed as an assessment tool to measure intercultural competence levels in order to identify specific orientations in participants that range from a monocultural to a more global mindset (Bennett 2004).

Few other researchers have studied cultural competence in health care professionals using the IDI®; only two studies of this nature are relevant to the project presented here. One used the IDI® as an assessment tool to gather information to facilitate development of a multidisciplinary course in cultural competence (Munoz et al. 2009). The other study demonstrated a more sustained model that utilized the IDI® as a pre-test, subjected participants to five months of cultural competence intervention, and then repeated the IDI® as a post-test (Altshuler et al. 2003). Synthesizing the applications of the IDI® from these findings, it is clear that this instrument can function both as an assessment and evaluation tool. Building on this foundation, a more deliberate use of the IDI® for assessment and evaluation can assist in both curriculum planning and intervention design.

This study reports on the efforts of one Midwestern university that developed a program, “Discover the Nurse Within”, based on the IDI® assessment. The various strategies implemented in this program contributed to an increase in the racial and/or ethnic diversity of the undergraduate student nurse population from 13% in 2003 to 29% in 2009. However, retention of students to matriculate to junior and senior coursework and subsequent graduation has been less successful. This may suggest a disconnect between the College of Nursing curriculum plan and the needs of students from diverse backgrounds. The purpose of this study was to identify the cultural competency of students, faculty, and staff as a first step in developing appropriate interventions to improve retention and graduation rates.

### Theoretical framework

Bhawuk and Brislin (1992) suggested, “To be effective in another culture, people must be interested in other cultures, be sensitive enough to notice cultural differences, and then also be willing to modify their behavior as an indication of respect for the people of other cultures” (p 416). Intercultural competence and understanding is central to improving relations across cultures (Bennett 1993; Hammer 2003). Intercultural competence is also necessary for domestic intercultural relations related to age, gender, ethnicity, and sexual orientation (Andrews et al. 2010).

The theoretical framework that grounds this study is the Intercultural Development Continuum which ranges from a monocultural mindset at one side of the continuum to an intercultural mindset at the other (Hammer 2009). It is based on the Developmental Model of Intercultural Sensitivity originally proposed by Bennett (2004) that consists of five orientations: Denial, Polarization, Minimization, Acceptance, and Adaptation.

**Figure 1:**
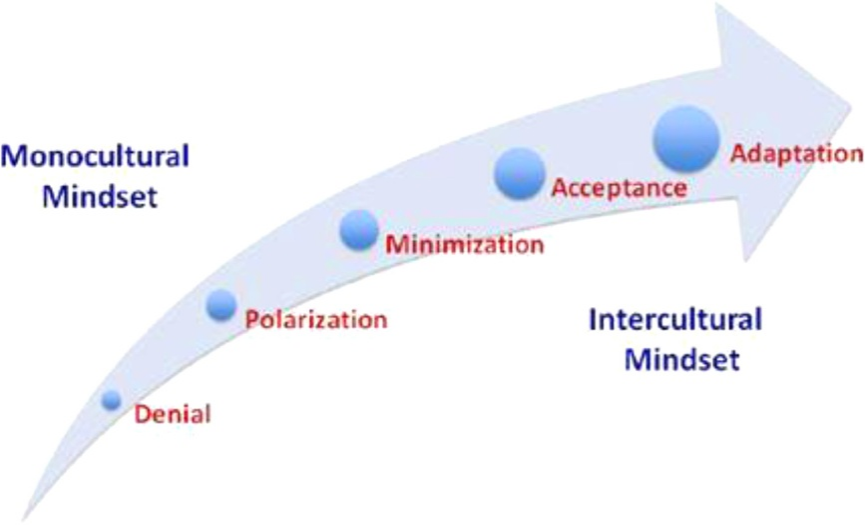
**The Intercultural Development Continuum.** Reproduced from the Intercultural Development Inventory Resource Guide by permission of the author, Mitchell R. Hammer, Ph.D., IDI, LLC. Copyright 1998, 2003, 2007, 2012 Mitchell R. Hammer, IDI, LLC. All Rights Reserved.

Denial is one of the developmental stages at the monocultural mindset side of the developmental continuum. A person in the Denial stage of development may demonstrate little identification of more complex cultural differences between himself/herself and people from different cultural backgrounds and assume that “others are like me”. Polarization has been defined as a judgmental orientation where a person uses an “us” versus “them” approach when interacting with people from different cultural backgrounds. A person in this stage notices cultural difference and often judges this difference negatively. Polarization may include a viewpoint where “my way” is better (defense) or “their way” is better (reversal). Minimization is the developmental stage where a person values commonalities between herself/himself and those from other cultural backgrounds; however there is little prominence placed on cultural differences. People in this stage of development may act upon the assumption that similarities are more important than differences and may even fail to recognize subtle differences that effect how a person’s behaviors could be interpreted. Acceptance is the designation for the stage where people have the ability to recognize cultural differences between their own culture and people from different cultural backgrounds and act in ways that communicate and appreciate difference. Finally, at the Adaptation level of development, people are operating with an intercultural mindset and are able to transfer cultural perspectives as well as adapt their behavior to various cultural contexts (Figure 1) (Hammer 2009).

The Aims of this study are as follows:

**Aim 1:** To examine the range of cultural developmental orientations of College of Nursing faculty and staff.

**Aim 2:** To examine the range of cultural developmental orientations of College of Nursing undergraduate and graduate students.

Research question: (1) Is there a difference in perceived orientation scores (PO) and developmental orientation (DO) scores for undergraduate versus graduate students, females versus males, and ethnic minority versus non-minority?

Research question: (2) Is there a difference in perceived orientation scores (PO) and developmental orientation (DO) scores related to age categories (18–21, 22–30, 31–40, 41–50, and 51–61 years) and time abroad categories (never, < 1 month-11 months, 1–5 years, 6–10 years, and > 10 years)?

The motivation for examining these variables arose from intercultural interactions observed in the College of Nursing. Anecdotal evidence suggested that age and time abroad may be important variables in evaluating an individual’s placement on the continuum. The demographic information collected through the IDI® allowed this hypothesis to be tested.

## Methods

### Design and sample

This study was part of a larger interventional study to enhance cultural development of the College of Nursing faculty, staff, and students. The sample for this study included 314 participants (students, faculty, and staff) in phase one of the parent study. Phase one included the initial administration of the IDI® as a pre-test to several cohorts of students over a two year period. Phase two includes the implementation of cultural development interventions with a post-test IDI® survey and is currently in process.

This prospective longitudinal cohort study commenced in fall 2010. There were 434 participants who were invited to partake in the study over a two year period including 397 students and 37 staff, part-time faculty, and full-time faculty (including administrators). Eligible research participants included all first-year, first-semester undergraduate and graduate nursing students, all full- and part-time faculty, and all full- and part-time staff in the College of Nursing. The final sample included 237 undergraduate and 40 graduate students as well as 37 faculty and staff for a response rate a 70% and 100% respectively. Participants who completed all aspects of the study were included in the analysis. Participation in this study was voluntary with no compensation; however, students were required to complete the IDI® or another equivalent assignment as a course requirement. The project received approval from the Lourdes University Institutional Review Board (IRB) where the research was conducted.

### Procedure

There was a four-part procedure for all groups involved in this study. The primary investigator visited all first-year, first-semester undergraduate and graduate student classrooms to explain and introduce the IDI® survey process. This introduction involved a brief explanation of what the IDI® is, a rationale for the study, and description of IRB-related human subject rights. All student questions regarding the study were answered at this time. The students then received an e-mail the next day from the primary investigator, which again invited them to participate in the study, and also included a personalized user name and password as well as the electronic survey link. Students had approximately two weeks to complete the survey. Then the primary investigator returned to the classroom to present aggregate IDI® survey results towards the end of the semester. The last phase of the process involved an individual feedback session where each student was given the opportunity to receive her/his IDI® results with a qualified administrator who was not the student’s current faculty member. Qualified administrators are faculty members who participated in a two and a half day seminar related to IDI® theory and how to administer the IDI®.

The study procedure for faculty and staff included an introduction of the study at a mandatory faculty meeting by the primary investigator who explained what the IDI® was, provided a rationale for the study, and informed faculty/staff of IRB related human subject rights. The primary investigator also sent an e-mail invitation to all full- and part-time faculty and staff that contained the same information provided at the faculty meeting. An outside consultant who was a qualified administrator was then brought to campus to administer the IDI® and present individual faculty and staff results. In addition, the consultant provided a three hour presentation regarding “Developing Intercultural Understanding” and also presented aggregate survey results. Due to confidentiality of the faculty and staff data, a basic report was given to the primary investigator that did not include raw data.

### Measures

The IDI® is a 50-item, electronically-administered instrument that measures intercultural development and is based on the Intercultural Development Continuum. The individual results of the IDI® readily allow viewing of the placement on the Intercultural Development Continuum in the following categories: Denial, Polarization (reversal or defense), Minimization, Acceptance, and Adaptation and assesses cultural development from a monocultural mindset at one end of the continuum to an intercultural mindset at other end of the continuum (Figure 1).

IDI® results reveal information related to perceived orientation (where participants believe they fall on the continuum) as well as developmental orientation (where participants actually fall on the continuum). The IDI® is considered a reliable and valid tool (Hammer 2003, 2009, 2011). Validity testing included a confirmatory factor analysis which resulted in the five aforementioned categories. The alpha coefficient reliabilities of the subscales include: Perceived Orientation (PO) Scale (.82), Developmental Orientation (DO) Scale (.83), Denial Subscale (.66), Defense Subscale (.72), Reversal Subscale (.78), Minimization Subscale (.74), Acceptance Subscale (.69), and the Adaptation Subscale (.71) (Hammer 2011). The confidential nature of the IDI® tool does not allow for the calculation of alpha coefficients for each of the subscales.

### Data analysis

All available raw data were analyzed using the Statistical Package for the Social Sciences (SPSS) 17.0. Selected components of the raw data for faculty and staff were not available since an expert consultant collected the data for faculty to promote anonymity. Analysis of the faculty and staff data utilized aggregate basic descriptive statistics as well as perceived and developmental orientations. The student data analysis included all independent variables and the dependent variables and had the following baseline analyses: basic descriptive statistics, assessment of missing data, and normal probability plots of the distribution. Independent-samples t-test and analysis of variance (ANOVA) were utilized to examine any significant mean differences in designated groups based on the research aims and questions. The significance level for all analyses was set *a priori* at ≤ .05.

## Results

Demographic data related to the program participants is noted in Table 1. In terms of diversity, the faculty and staff appeared to be a homogeneous group with the majority of the population of the female gender and only 8% of participants from a racial and/or ethnic minority group. The student population (both undergraduate and graduate students) is more diverse with 14% of the male gender and 20% from a racial and/or ethnic minority group.

**Table 1:** **Demographics of faculty and staff (N = 37) and students (N = 277)**

	Faculty & staff		Students	
Characteristic	n	Percent (%)	n	Percent (%)
Gender				
Female	36	97	238	86
Male	1	3	39	14
Age Category				
18-21	0	0	50	18
22-30	5	13	102	37
31-40	5	13	69	25
41-50	7	17	39	14
51-60	12	33	17	6
61 and over	8	24	0	0
Total Amount of Time Lived in Another Country				
Never Lived in Another Country	32	87	227	82
< 3 months	3	7	11	4
3-6 months	1	3	3	1
7-11 months	0	0	3	1
1-2 years	0	0	9	3
3-5 years	1	3	9	3
6-10 years	0	0	6	2
> 10 years	0	0	9	3
World Region Lived During Formative Years (to age 18)				
North America	37	100	262	95
Central America	0	0	3	1
Middle East	0	0	3	1
Africa	0	0	3	1
Asia Pacific	0	0	3	1
Other	0	0	3	1
Ethnic Minority in Your Country (Yes)	3	8	55	20
Education Level				
High School Graduate	3	7	111	40
Post Secondary University Graduate	8	21	130	47
M.A. Degree or Equivalent Graduate Degree	17	48	6	2
Ph.D. Degree or Equivalent Graduate Degree	9	24	0	0
Other	0	0	30	11
Current Position at Institution (Faculty & Staff)				
Administration	6	17	N/A	N/A
Faculty	19	50	N/A	N/A
Staff	11	30	N/A	N/A
Other	1	3	N/A	N/A
Students				
Undergraduate	N/A	N/A	237	86
Graduate	N/A	N/A	40	14

**Figure 2:**
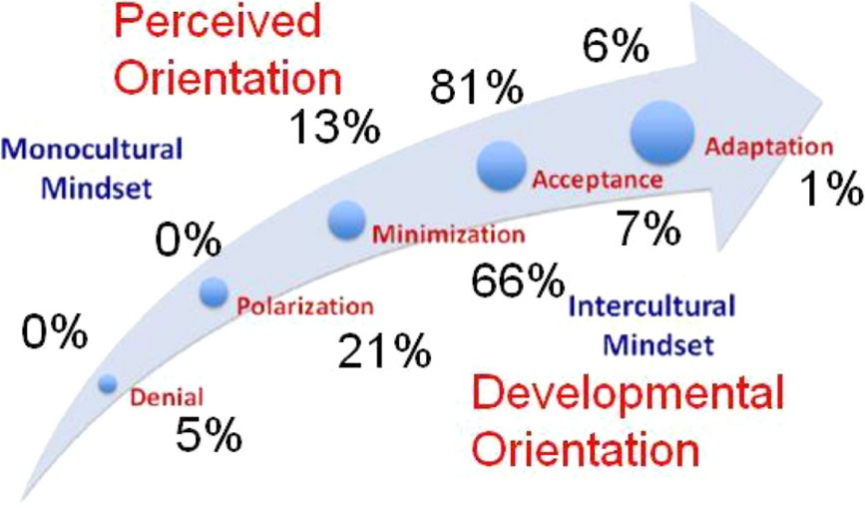
**Perceived and developmental orientation scores for undergraduate and graduate students.** Perceived orientation: Minimization *n =* 36, Acceptance *n =* 224, Adaptation *n =* 17. Developmental orientation: Denial *n =* 14, Polarization *n =* 58, Minimization *n =* 183, Acceptance *n =* 19, and Adaptation *n =* 3.

**Figure 3:**
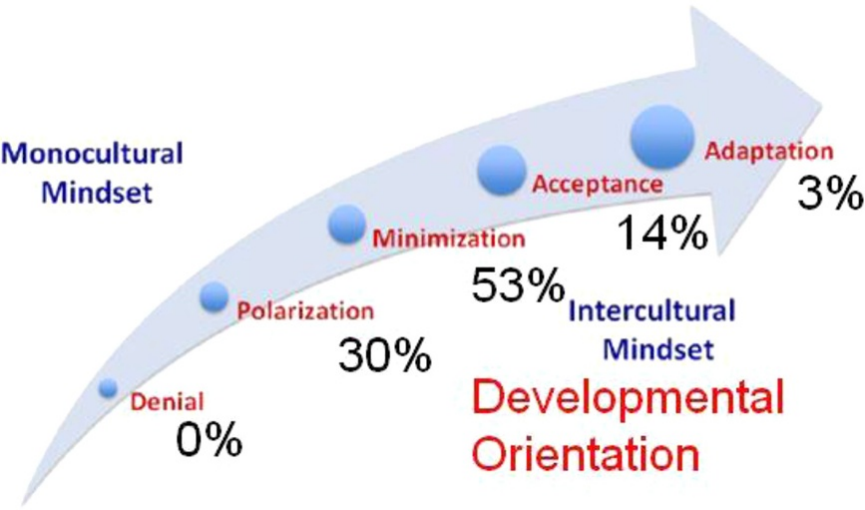
**Developmental orientation scores for faculty and staff.** Developmental orientation: Polarization *n =* 11, Minimization *n =* 20, Acceptance *n =* 5, and Adaptation *n =* 1.

IDI® results in terms of perceived and developmental orientations for students, faculty, and staff are detailed in Figures 2 and 3. As noted from the figures, there were more faculty and staff in the Acceptance and Polarization developmental categories as compared to students whereas students had a greater number of individuals scoring in the Minimization category as compared to faculty and staff. Faculty and staff had no participants who scored in Denial and students had very few people scoring at Adaptation. Another compelling finding was that 98% of student participants overestimated their level of cultural competency. The data for orientation gap scores and remaining t-test and ANOVA testing were completed only for students because data were not available for faculty and staff.

Table 2 displays the results of an independent-samples t-test that was conducted to explore differences in perceived and developmental orientations by student type, gender, and ethnicity. As one may note from the table, there were no statistically significant differences between undergraduate and graduate students or differences related to gender. There were however, statistically significant differences in perceived and developmental orientation scores for ethnic minorities compared to ethnic non-minorities. The eta-squared values for both perceived and developmental orientations were small (.01) in both cases.

**Table 2:** **Differences in perceived and developmental orientations by student type, gender, and ethnicity**

Variable	Perceived orientation					Developmental orientation				
	M	SD	t	df	p	M	SD	t	df	p
Student Type			0.11	275	.91			−0.34	275	.73
Undergraduate (*n =* 237)	121.85	5.88				95.16	15.25			
Graduate (*n =* 40)	121.74	4.97				96.03	12.43			
Gender			0.18	273	.86			−0.41	273	.68
Male (*n =* 38)	121.98	6.51				94.35	16.96			
Female (*n =* 237)	121.80	5.62				95.41	14.51			
Ethnicity			1.99	272	.05			1.97	272	.05
Minority (*n =* 55)	123.22	5.65				98.85	14.21			
Non-Minority (*n =* 219)	121.50	5.75				94.46	14.96			

A one-way, between-groups ANOVA was conducted to explore the differences in perceived and developmental orientation scores among five age categories of students (18–21, 22–30, 31–40, 41–50, and 51–61 years). There was a statistically significant difference in perceived orientation scores for the five age groups: F (4, 260) = 2.48, p = .04. Even though the number reached statistical significance, the actual difference in mean scores between the groups was small as evidenced by an eta-squared value of .04. Post-hoc comparisons using Least Significant Difference (LSD) test indicated that the mean score for 18–21 years was significantly lower (M = 119.94, SD = 5.76) than 22–30 years (M = 122.04, SD = 5.59) and 31–40 years (M = 123.27, SD = 5.84). Ages 41–50 years (M = 121.72, SD = 6.26) and 51–61 (M = 120.80, SD = 4.64) did not differ significantly from the other groups. In terms of developmental orientation and age, there was no statistically significant difference in scores for the five age groups: F (4, 260) = 2.28, p = .06.

A one-way ANOVA was conducted to explore the differences in perceived and developmental orientation scores among time-abroad categories (never, < 1 month-11 months, 1–5 years, 6–10 years, and >10 years). Both perceived orientation scores F (4, 270) = 2.06, p = .09 and developmental orientation scores F (4, 270) = 1.42, p = .23 were not statistically significant for differences between groups.

## Discussion

IDI® results in the general population tend to follow a bell-curve or normal distribution. Therefore, approximately 68% of the general population will fall within the Minimization category, 28% will fall in the Polarization and Acceptance categories, and 4% at the tail ends of the Denial and Adaptation categories (IDI® training June 2011). The results of this study reflect trends that faculty, staff, and students in the College of Nursing in this case study represent the general population. A follow up post-test will help to answer the question of whether the program strategies for cultural development and competence may in part contribute to improvements in intercultural competence.

The IDI® as an assessment tool assists in the understanding of cultural development at both the individual and the aggregate levels. At the individual level, participants in this study met with an IDI® qualified administrator and were encouraged to reflect on their results and consider the significance of their results in terms of further cultural development. At the aggregate level, the IDI® results provided a representation of cultural development within the College of Nursing. Study data indicate that two approaches may enhance faculty confidence and self-efficacy related to the interaction of faculty with diverse students. The first is continuing to embed intercultural experiences throughout the curriculum, and the second is skill building for faculty in the area of culturally competent teaching strategies. In addition, greater attention to the development of a more global mindset throughout the curriculum may advance students through the intercultural developmental continuum.

The results of this study demonstrate that self-perception is not an adequate measure of cultural competency. Only 2% of the student participants in this study were accurate in their cultural self-assessment. Therefore, it is vital to compare perceived orientation and developmental orientation to have a holistic view of a subject’s intercultural mindset.

Study results also revealed statistically significant differences in perceived and developmental orientation scores for ethnic minorities compared to ethnic non-minorities. Minority students had higher developmental scores (M = 98.85, SD = 14.21) than non-minority students (M = 94.46, SD = 14.96), which is consistent with Kim’s (2005) work regarding ethnic group strength. According to Hammer (2009), IDI® results may be generalizable across cultural groups. In theory, ethnic minority and non-minority results should not differ however, the current study did not support this notion. It may be postulated the reason for the statistically significant higher scores for the minority students in this study is that these students not only interact with the members of their own cultural groups, but that they are also continuously exposed and navigate within cultures of others.

It was also postulated that there would be a difference in PO and DO scores according to age and time abroad. In terms of age, PO scores were lower for students aged 18–21 when compared to those students aged 22–30 and 31–40. The 18–21 year old students may have perceived that they had less cultural experience because of lack of intercultural exposure and life experience and/or experiences related to travel abroad. In terms of DO, there were no differences according to age.

Additionally, there were no statistically significant differences in PO and DO scores in relation to travel abroad experiences. These results may be explained by low participant numbers in the 6–10 years and greater than 10 years abroad categories. It is also important to note that time abroad does not necessarily equate to cultural competence. In other words, in this study, the greater the number of years abroad did not necessarily mean greater cultural competence. Therefore, if someone has spent several years abroad and is still at a monocultural mindset, perhaps guided opportunities may help with individual growth and cultural development. Perhaps guided cultural immersion activities that occur in a home country versus internationally would be similarly effective at developing someone culturally.

Therefore, when planning travel abroad experiences for college students, it is important to consider a comprehensive orientation program that includes cultural general and specific training as well as immersion experiences. When planning meaningful educational travel, Cushner (2004) recommends, “One must point out for participants the processes involved in cultural learning: understanding themselves as cultural beings; becoming less ethnocentric in their orientation; unlearning some of what they have spent their entire lives learning; and the relearning of new knowledge, insights, and skills that will enable them to function effectively with a new mindset and within a new context” (p 16–17).

### Strengths, limitations, and future research

The strength of this research includes the thoroughness of the data collection processes, the relatively large sample size of students, and the appropriate utilization of consultants as well as access to the consultant who developed the tool. In addition, this instrument may be useful for other colleges and universities to measure cultural development. A major strength of this tool is that it not only measures how culturally competent a person thinks he/she are but it also assesses how competent she/he actually is. The aggregate results of this study indicate that there was a mismatch between perception and reality.

Limitations of this study include the fact that some of the raw faculty and staff data to conduct a more in-depth analysis was not available. Additionally, this study did not focus on the qualitative data that is obtained with the IDI® (e.g. description of cultural situations that the person perceived ended positivity and/or negatively). The latter limitation is a potential area for future research.

In addition, self-reflection has been noted to be a means for personal growth that leads to deep learning (Emery 2012; Patil 2013), and the IDI® offers the opportunity to recognize areas of personal growth in terms of cultural development. A future longitudinal study could compare results of pre- and post-IDI® results after a curriculum intervention to determine its efficacy.

Overall, the IDI® was a valuable self-reflection tool to assess cultural development. At the individual level, it has allowed for self-reflection and awareness to the reality of cultural development, attitudes, and values. At an institutional level, the aggregate results provided a framework to assist in the examination of department policies, procedures, faculty development opportunities, and curriculum design with the ultimate goal of graduating a more culturally competent nursing workforce to serve the greater community.
